# Uptake of mandated pregnancy warnings in the Australian alcoholic ready‐to‐drink beverage market

**DOI:** 10.1111/dar.13758

**Published:** 2023-10-11

**Authors:** Bella Sträuli, Tazman Davies, Stephen Jan, Leon Booth, Nadia Laznik, Fraser Taylor, Simone Pettigrew

**Affiliations:** ^1^ The George Institute for Global Health UNSW Sydney Sydney Australia

**Keywords:** alcohol, health promotion, labelling, pregnancy, prevention

## Abstract

**Introduction:**

A mandatory pregnancy warning was introduced in Australia 2020 to advise the public of the potential harms of prenatal alcohol exposure. Due to industry pressure, a 3‐year implementation period was granted. The aim of this study was to analyse the extent to which the mandatory warning had been applied to ready‐to‐drink (RTD) alcohol product labels almost 2 years into the implementation period.

**Methods:**

The sample included 491 RTD products sold in three alcohol stores in Sydney, Australia in March–May 2022. Identified warnings were categorised as a mandated warning, a DrinkWise warning (an industry‐developed option) or ‘Other’ warning. Analyses were conducted overall and by RTD type.

**Results:**

Almost all (94%) of the sampled RTD products had some form of pregnancy warning, but only 36% displayed the mandatory version. Of the non‐mandatory warnings, 74% were DrinkWise warnings (42% of total sample) and 27% were ‘Other’ warnings (15% of total sample). There was no apparent relationship between alcohol content and likelihood of displaying a mandatory warning.

**Discussion and Conclusions:**

Two years into the three‐year implementation period for the mandatory pregnancy warning, only around one‐third of the assessed RTD products exhibited compliance. Uptake of the mandatory pregnancy warning appears to be slow. Continued monitoring will be required to determine whether the alcohol industry meets its obligations within and beyond the implementation period.

## 1 INTRODUCTION

Alcohol is a teratogen that can cause serious harm such as fetal alcohol spectrum disorder, premature birth, low birthweight and adverse obstetric outcomes for the mother [1, 2]. Pregnancy warnings on alcohol products can increase consumer awareness of the risks associated with drinking while pregnant, and therefore have the potential to reduce fetal and maternal harm from alcohol [3]. More than 30 countries have implemented such warnings to date [4], including Australia where a new pregnancy warning (hereafter called ‘mandatory warning’) was introduced by Food Standards Australia New Zealand in July 2020, with a three‐year implementation period granted until July 2023 [5].

The introduction of this warning had a long gestation period. Australian health organisations have been advocating for mandatory pregnancy warnings for more than 20 years [6], and in 2011 a major food and beverage labelling review (‘Labelling Logic’) recommended the introduction of mandatory pregnancy warnings on alcohol products [7]. However, the alcohol industry's social aspects/public relations organisation, DrinkWise, was permitted to introduce its own voluntary pregnancy warning system instead. This system comprised three designs, typically presented in greyscale, featuring the DrinkWise logo and either: (i) a pictogram of a struck‐through silhouette of a drinking pregnant woman; (ii) pregnancy‐related text (e.g., “It is safest not to drink while pregnant”); or (iii) combined pictogram and text [8]. Nearly a decade after the Labelling Logic review recommendation, the mandatory system was introduced because the voluntary system had failed to achieve sufficient market coverage [3].

There are two versions of the mandatory warning that apply to beverages containing >1.15% alcohol by volume. The version applicable to most products is the black, red, and white ‘warning mark’, which includes a struck‐through silhouette of a drinking pregnant woman, accompanied by the text ‘PREGNANCY WARNING: Alcohol can cause lifelong harm to your baby’ [9]. For individual products under 200 mL in size, a version showing just the pictogram can be used. During development, these warning formats underwent consumer testing to ensure their salience and effectiveness with target audiences [3].

The new mandatory pregnancy warning is an important public health initiative given around half of pregnant women in Australia consume alcohol before they are aware they are pregnant, and 15% of pregnant women continue consuming alcohol post‐confirmation [10]. It is estimated that around 2% of Australian babies are born with some degree of fetal alcohol spectrum disorder [11, 12], however, this may be an underestimate given higher rates found in comparable countries (e.g., [13]). Women can experience social pressure to consume alcohol during pregnancy [14], and thus warning labels also have an important role to play in raising awareness among members of the broader community who can support women to be alcohol‐free during pregnancy [15]. In this context, it is critical for the mandatory warning to be adopted by the alcohol industry as quickly as possible.

To obtain insight into alcohol producers' commitment to introducing the mandated pregnancy warning in a timely fashion, the aim of the present study was to assess uptake in the ready‐to‐drink (RTD) market almost 2 years into the three‐year implementation period. RTDs were chosen because they constitute the fastest‐growing drinks category by volume, especially hard seltzers which have a projected increase of 24% between 2020 and 2025 in Australia [16]. Given the dynamic nature of this market, it was deemed most likely to have had the opportunity to include the new pregnancy warning on product labels. RTDs are defined as beverages that include both alcoholic (e.g., vodka) and non‐alcoholic (e.g., soft drink) components, pre‐combined and ready for immediate consumption [17].

## 2 METHODS

The information contained on alcohol product labels was collected according to an established protocol used to document nutrition information on food packaging (the FoodSwitch protocol [18]). In March–May 2022, data collectors visited three inner‐city stores representing three major alcohol retailers in Sydney, Australia (Dan Murphy's, Liquorland and BWS). Photographs were taken of products available for sale to capture label content.

The product‐ and health‐related information captured in the photographs was subsequently extracted and coded. For the present study, analyses focused on the presence and type of pregnancy warning information displayed on RTD products in the data set (*n* = 491). The pregnancy warning information was categorised as being presented in the following formats: either of the two mandatory warning options (warning mark or pictogram‐only), any of the three DrinkWise options (DrinkWise logo + text only, DrinkWise logo + pictogram only, DrinkWise logo + text + pictogram) or two ‘Other’ warning variations (pictogram‐only, pictogram + text). This coding system reflects the history of pregnancy warning labels in Australia whereby there were initially no requirements, and any kind of warning could be used (or not), followed by the introduction of the voluntary DrinkWise system, finally culminating in the introduction of the mandatory warning.

Products were categorised according to base alcohol types, such as gin, vodka or wine. An exception was the hard seltzer products, which were most commonly mixed with a neutral alcohol base (made of fermented grain, malt or cane sugar) and sparkling water. Those with more than one type of alcohol present were classified as multiple. All remaining products were designated as ‘other’. A logistic regression was used to test the relationship between alcohol by volume percentage and presence of a mandatory warning. Additional methodological information is provided in Table S1, Supporting Information.

## 3 RESULTS

The proportions of RTD products displaying pregnancy warnings by alcohol type are shown in Table 1. Most of the assessed products (94%) displayed a pregnancy warning of some kind, but only 36% featured one of the mandatory warning options. The pictogram‐only version of the mandatory warning was rare (1 product, 0.2%), which was consistent with the proportion of small products in the dataset that were eligible to use this version.

**TABLE 1:** Presence of pregnancy warnings on sampled ready‐to‐drink alcoholic beverages.

Product type	Number of products, *n*	Average ABV%, %	Any warning, *n* (%)		Mandatory warning, *n* (%)	
All products	491	6.3	462 (94%)		178 (36%)	
Hard seltzer	135	4.5	121 (90%)		55 (41%)	
Vodka	121	5.9	115 (95%)		33 (27%)	
Whisky	85	7.5	85 (100%)		39 (46%)	
Gin	60	6.2	56 (93%)		21 (35%)	
Rum	43	7.1	39 (91%)		17 (40%)	
Multiple^a^	24	11.3	23 (96%)		7 (29%)	
Wine	13	8.3	13 (100%)		1 (8%)	
Other^b^	6	6.5	6 (100%)		3 (50%)	
Tequila	4	9.1	4 (100%)		2 (50%)	

Abbreviation: ABV, alcohol by volume.

^a^ Products with multiple alcohols combined.

^b^ For example, products containing liqueurs.

For the whisky, wine, tequila and ‘other’ RTD types, all products had some form of pregnancy warning. The category of products least likely to display a pregnancy warning was hard seltzers (90%). Use of a mandatory warning was highest among tequila (50%), ‘other’ (50%) and whisky (46%) RTD products, and lowest for wine (8%), vodka (27%) and multiple alcohol (29%) RTD products.

The alcohol‐by‐volume percentage results overall and by product subcategory are shown in Table 1. Average alcohol content was 6.3%, ranging from 4.5% for hard seltzers and 11.3% for products with multiple alcohol types. There was a non‐significant relationship between alcohol content and mandatory warning presence (*B* = −0.03, *p* > 0.05).

Of the 284 products displaying non‐mandatory pregnancy warnings, 74% (43% of the total sample) used DrinkWise versions and 27% were ‘Other’ warnings (15% of the total sample) (see Table 2). Most of the non‐mandatory warnings featured a pictogram (93%; 81% pictogram only), with few including both a pictogram and text (12%).

**TABLE 2:** Presence of variations of DrinkWise and ‘Other’ warnings on sampled ready‐to‐drink alcoholic beverages.

Product type	Total, *n*	Non‐mandatory pregnancy warning labels (*n* = 284)				
		DrinkWise (74%)			Other (27%)	
		Text, *n* (%)	Pictogram, *n* (%)	Text + pictogram, *n* (%)	Pictogram, *n* (%)	Text + pictogram, *n* (%)
All products	284	22 (8%)	159 (56%)	27 (10%)	71 (25%)	5 (2%)
Vodka	82	5 (6%)	41 (50%)	6 (7%)	30 (37%)	0 (0%)
Hard seltzer	66	1 (2%)	41 (62%)	6 (9%)	18 (27%)	0 (0%)
Whisky	46	12 (26%)	14 (30%)	11 (24%)	9 (20%)	0 (0%)
Gin	35	0 (0%)	23 (66%)	3 (9%)	6 (17%)	3 (9%)
Rum	22	2 (9%)	15 (68%)	0 (0%)	5 (22%)	0 (0%)
Multiple^a^	16	1 (0%)	10 (63%)	1 (6%)	2 (0%)	2 (0%)
Wine	12	1 (8%)	10 (83%)	0 (0%)	1 (8%)	0 (0%)
Other^b^	3	0 (0%)	3 (100%)	0 (0%)	0 (0%)	0 (0%)
Tequila	2	0 (0%)	2 (100%)	0 (0%)	0 (0%)	0 (0%)

*Note*: DrinkWise pregnancy warnings were identified by the presence of the DrinkWise logo adjacent to the pregnancy warning.

^a^ Products with multiple alcohols combined.

^b^ For example, products containing liqueurs.

Across all products sampled, 4% displayed text only, 47% displayed a pictogram only and 43% displayed both (results not shown). The largest proportion of products displaying a warning that included both a pictogram and text was found for whisky (59% of all whisky products) and the lowest proportion was for wine products (8%).

## 4 DISCUSSION

This study examined the prevalence of pregnancy warning labels on RTD alcoholic beverages almost 2 years into the three‐year implementation period of Australia's mandatory pregnancy warning system. Although the overall prevalence of pregnancy warnings on the sampled products was high (94%), only around one‐third (36%) displayed a mandatory version.

Previous research has shown that alcohol product warnings including both a pictogram and text are more effective than warnings including just one of these two components [19]. This is reflected in the design of the mandatory pregnancy warnings [9] but is not uniformly characteristic of the non‐mandatory variations. The results of the present study suggest that where industry can choose a warning type, they typically preference the pictogram‐only variation, which is likely to be attributable to its smaller footprint. It is therefore important to mandate combined formats and monitor their use.

The primary study limitation was the collection of data from just three stores, although these represented three of the largest alcohol chains in Australia. All three stores were from inner‐city Sydney, and product ranges could vary in other geographical contexts. Second, the focus on a single product category (RTDs) precludes generalisation of the results to the broader alcohol market. The average alcohol by volume percentage range identified across the RTD category was reasonably wide (4.5–11.3%), but does not account for products with much higher alcohol contents (e.g., spirits). Further research is therefore needed to determine the extent of pregnancy warning uptake across all alcoholic beverages, especially at the end of the implementation period to assess compliance.

Two years post‐introduction of the new Australian pregnancy warning, only around one‐third of the assessed RTD products displayed the mandatory pregnancy warning. Effective warning labels are a simple tool that can raise awareness and act as a reminder of the potential harms to the fetus and the mother associated with pre‐natal alcohol exposure. It is therefore critical that the alcohol industry intensifies its efforts to ensure compliance with the mandatory requirement.

## AUTHOR CONTRIBUTIONS

Each author certifies that their contribution to this work meets the standards of the International Committee of Medical Journal Editors.

## FUNDING INFORMATION

This study was funded by National Health and Medical Research Program Grant (#GNT1149987). The funder had no role in determining the study design, the contents of the paper, nor decisions relating to its publication.

## CONFLICT OF INTEREST STATEMENT

The authors declare no conflicts of interest.

## Supporting information


**Table S1.** Examples of pregnancy warning information provided on alcoholic beverages in Australia.
