# Supplementary material for: Uptake of mandated pregnancy warnings in the Australian alcoholic ready‐to‐drink beverage market
Source: Drug Alcohol Rev. 2023 Oct 11;43(1):165–9. doi: 10.1111/dar.13758 (PMC10953340; doi:10.1111/dar.13758)
Supplement: Supplementary file 1 — Table S1. Examples of pregnancy warning information provided on alcoholic beverages in Australia. [file DAR-43-165-s001.docx]

Table S1. Examples of pregnancy warning information provided on alcoholic beverages in Australia

| Mandatory pregnancy warning label [1] | **Description:** The mandatory pregnancy warning labels come in two variations that include the colours black, white and red: (i) the ‘mark’ that includes a pictogram comprising a silhouette of a pregnant woman with a strikethrough, accompanied by the text ‘PREGNANCY WARNING: Alcohol can cause lifelong harm to your baby’; and (ii) only a pictogram comprising a silhouette of a pregnant woman with a strikethrough. The pictogram is only permitted to be used on beverages <200 mL. | |
| --- | --- | --- |
|  | Mark | 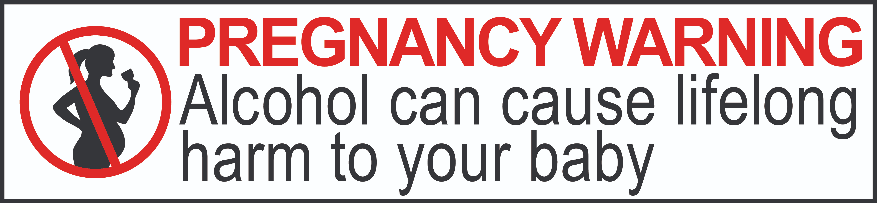 |
|  | Pictogram | 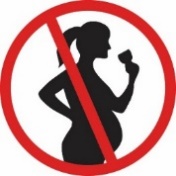 |
| DrinkWise pregnancy warning label [2] | **Description:** DrinkWise have had three primary forms of pregnancy labels: (i) the ‘mark’ which included a pictogram of a silhouette of a pregnant woman with a strikethrough accompanied by pregnancy related text, most commonly “It’s safest not to drink while pregnant”; (ii) a pictogram of a silhouette of a pregnant woman with a strikethrough only; and (iii) pregnancy-related text message most commonly “It’s safest not to drink while pregnant”. Notably, these designs were most commonly greyscale but also came with green elements too (for example, the strikethrough was green). The DrinkWise labels are easily identifiable by their logo ‘Get the Facts DrinkWise.org.au’. Examples are shown below.  Note: These pregnancy warnings are no longer shown, provided or promoted for use on the DrinkWise website. | |
|  | Mark | 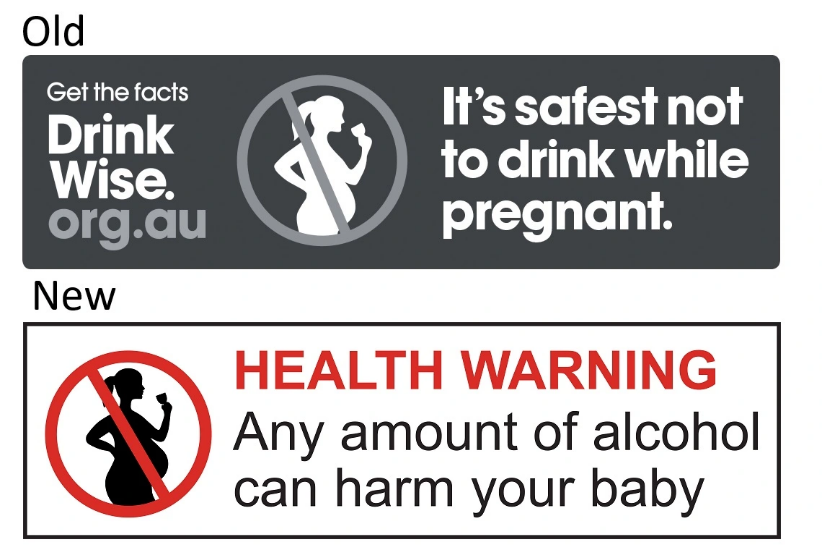 |
|  | Pictogram | 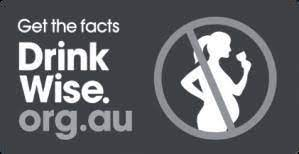 |
|  | Text | 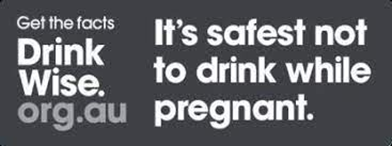 |
| ‘Other’ pregnancy warning label | **Description:** All other pregnancy warnings were classified as ‘other’, examples shown below. | |
|  | Mark | 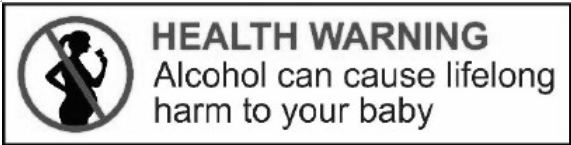 |
|  | Pictogram | 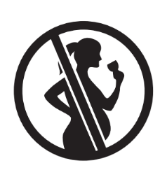 |

**References**

1. FSANZ. Pregnancy Warning Labels Downloadable files 2023. Available from: <https://www.foodstandards.gov.au/industry/labelling/Pages/pregnancy-warning-labels-downloadable-files.aspx>.

2. FSANZ, Pregnancy warning labels on packaged alcohol - A review of recent literature Proposal P1050. 2020. p. 1-77.

**Data collection and analysis**

Data collection was conducted in three alcohol stores in inner city Sydney:

- Dan Murphy’s inner city, Sydney – larger store, with approximately 5,000 products collected across all product categories
- Liquorland inner city, Sydney – medium-sized store with approximately 2,000 products collected across all product categories
- BWS inner city, Sydney – medium-sized store, with approximately 2,000 products collected across all product categories

Data collection was conducted by eight trained data collectors who used the established FoodSwitch protocol. This involved using a purpose build app that prompted the data collectors to take photographs of all label elements on each product. Information captured from alcohol labels included product type, brand name, manufacturer name, alcohol content, standard drinks per container, warnings, and claims. The photographs were then uploaded into a content management system accessible by a data entry team that systematically coded the label content.

For the present study, author XX (*blinded for review*) analysed the photographs of ready-to-drink (RTD) alcoholic beverages and compared against the initial coding to ensure accurate capture of the presence of pregnancy warning labels and to perform finer-level coding to distinguish between mandatory, DrinkWise, and ‘Other’ warnings (as per the definitions and examples provided in Supplementary Table S1).
